# Endothelial Cell Phenotypes Demonstrate Different Metabolic Patterns and Predict Mortality in Trauma Patients

**DOI:** 10.3390/ijms24032257

**Published:** 2023-01-23

**Authors:** Hanne H. Henriksen, Igor Marín de Mas, Lars K. Nielsen, Joseph Krocker, Jakob Stensballe, Sigurður T. Karvelsson, Niels H. Secher, Óttar Rolfsson, Charles E. Wade, Pär I. Johansson

**Affiliations:** 1 Section for Transfusion Medicine, Capital Region Blood Bank, Rigshospitalet, University of Copenhagen, 2100 Copenhagen, Denmark; 2 CAG Center for Endotheliomics, Copenhagen University Hospital, Rigshospitalet, 2100 Copenhagen, Denmark; 3 Novo Nordisk Foundation Center for Biosustainability, Technical University of Denmark, 2800 Kongens Lyngby, Denmark; 4 Australian Institute for Bioengineering and Nanotechnology (AIBN), The University of Queensland, 4072 Brisbane, Australia; 5 Center for Translational Injury Research, Department of Surgery, University of Texas Health Science Center, Houston, TX 77030, USA; 6 Department of Anesthesia and Trauma Center, Center of Head and Orthopedics, Rigshospitalet, 2100 Copenhagen, Denmark; 7 Center for Systems Biology, University of Iceland, 101 Reykjavik, Iceland; 8 Department of Anesthesiology, Centre for Cancer and Organ Diseases, Rigshospitalet, University of Copenhagen, 2100 Copenhagen, Denmark

**Keywords:** trauma, metabolomics, endotheliopathy, systems biology, genome-scale metabolic model, tricarboxylic acid cycle

## Abstract

In trauma patients, shock-induced endotheliopathy (SHINE) is associated with a poor prognosis. We have previously identified four metabolic phenotypes in a small cohort of trauma patients (N = 20) and displayed the intracellular metabolic profile of the endothelial cell by integrating quantified plasma metabolomic profiles into a genome-scale metabolic model (iEC-GEM). A retrospective observational study of 99 trauma patients admitted to a Level 1 Trauma Center. Mass spectrometry was conducted on admission samples of plasma metabolites. Quantified metabolites were analyzed by computational network analysis of the iEC-GEM. Four plasma metabolic phenotypes (A–D) were identified, of which phenotype D was associated with an increased injury severity score (*p* < 0.001); 90% (91.6%) of the patients who died within 72 h possessed this phenotype. The inferred EC metabolic patterns were found to be different between phenotype A and D. Phenotype D was unable to maintain adequate redox homeostasis. We confirm that trauma patients presented four metabolic phenotypes at admission. Phenotype D was associated with increased mortality. Different EC metabolic patterns were identified between phenotypes A and D, and the inability to maintain adequate redox balance may be linked to the high mortality.

## 1. Introduction

Trauma is a leading cause of death, and in the United States accounts for more than 79,000 deaths annually [1,2], more deaths than HIV, tuberculosis, and malaria combined [3]. The poor prognosis of trauma patients is associated with activation and dysfunction of the endothelial cell (EC) membrane by over-activation of the sympathetic nervous system releasing toxic levels of catecholamines, entitled shock-induced endotheliopathy (SHINE) [4]. SHINE results in extravasation and increased tissue pressure, microvascular thrombus formation leading to impaired oxygen delivery, multiple organ failure, and ultimately death [5,6,7,8].

Investigating the pathophysiological metabolic mechanism of EC in SHINE in vivo is not feasible since microvascular biopsy of EC in patients undergoing an acute critical state is difficult, if not impossible. Therefore, a biologically-validated, genome-scale metabolic model of the endothelial cell (EC-GEM) has been developed to circumvent this constraint [9]. EC-GEM is based on the high impact the EC metabolism has on plasma metabolic changes since the vascular compartment consists of more than 1 trillion EC in constant contact with the circulating blood [10]. Also, the link between the blood and the endothelium has been described as a single, dynamic organ system, rather than two separate systems [11]. This supports that metabolic changes in the plasma indicate the metabolic state of the EC. The intracellular endothelial metabolic pathways in SHINE have been inferred by integrating plasma metabolites from 20 trauma patients into EC-GEM [12]. Four metabolic phenotypes with differences in the intracellular response to SHINE were identified. Notably, there were different fluxes in the production of acetyl-CoA with an energetic shift from glycolysis towards β-oxidation observed in one of the phenotypes, labeled D. Phenotype D was characterized by hyperglycemia, increased epinephrine levels, and severe endothelial glycocalyx shedding [5,13].

Here, we investigated the inferred intracellular endothelial metabolism in a larger cohort of trauma patients to evaluate whether the four identified metabolic phenotypes were associated with 30-day mortality.

## 2. Results

### 2.1. Four Phenotypes in Trauma Patients

**Figure 1:**
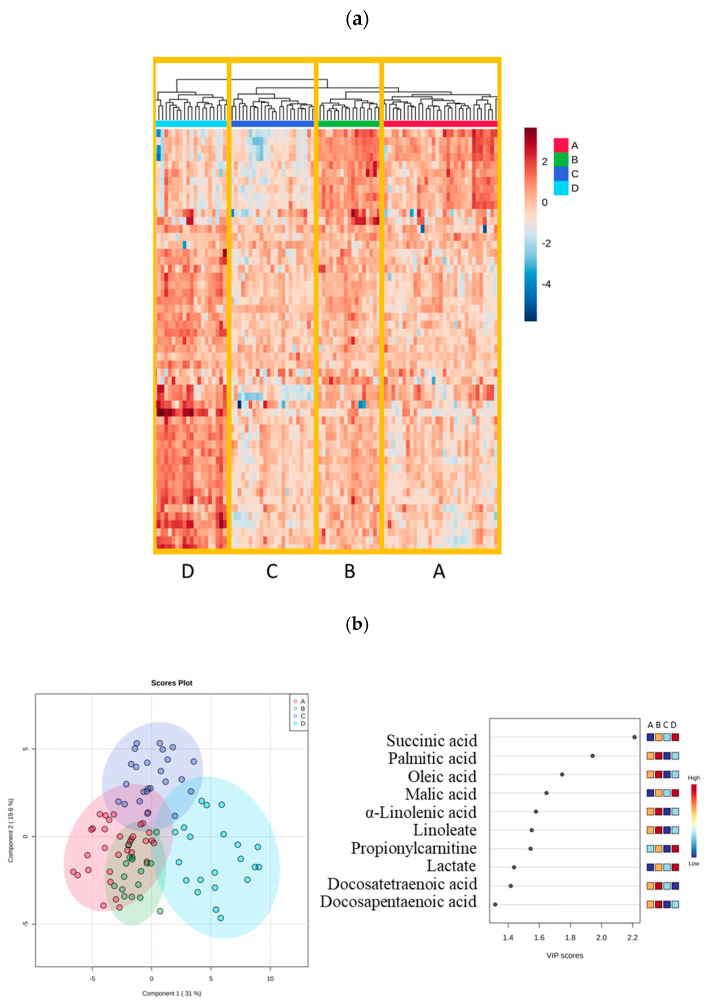
**Plasma metabolites in trauma patients demonstrated four shock-induced endotheliopathy phenotypes: A, B, C, and D.** (**a**) Heatmap combined with a dendrogram analysis using cluster algorithm for 54 quantified metabolites from plasma demonstrated four shock-induced endotheliopathy phenotypes: A, B, C, and D. (**b**) Partial Least-Squares Discriminant Analysis (PLS-DA) identifying the top-10 importance of the variables among the phenotypes.

The 95 trauma patients’ metabolic profiles revealed four plasma metabolic clusters; phenotypes A, B, C, and D, identified by a hierarchical clustering dendrogram (Figure 1a). Phenotype D had its own branch in the dendrogram, whereas phenotype C shared a top branch with phenotypes A and B but was further divided into its own branch next to phenotype D. Phenotypes A and B shared two branches at the dendrogram. The top-10 variables of importance in the PLS-DA plot used to best discriminate between the phenotypes were: succinic, palmitic, oleic, malic, α-linolenic docosatetraenoic, and docosapentaenoic acids, linoleate, propionyl carnitine, and lactate (Figure 1b). The top metabolite to discriminate between the four phenotypes, succinic acid, was notably elevated in phenotype D compared to the other phenotypes (*p* < 0.001).

### 2.2. Clinical Characteristics of the Four Phenotypes

**Figure 2:**
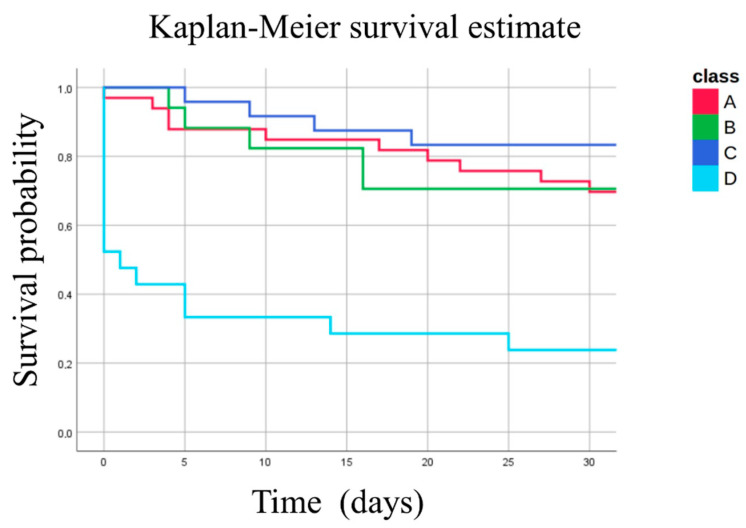
**Shock-induced endotheliopathy phenotypes mortality survival curve for 95 critically ill trauma patients. Survival curves of phenotypes A, B, C, and D**.

The clinical characteristics of the four phenotypes are presented in Table 1 and the survival curve in Figure 2. The patients belonging to phenotype D were seriously injured (higher ISS), received more transfusions, were more shocked, and had a higher mortality rate than patients from the other phenotypes.

### 2.3. Catecholamine and Endothelial Biomarkers

Phenotype D had higher levels of both epinephrine (*p* < 0.001) and norepinephrine (*p* < 0.001) compared to other members of the trauma cohort (Table 2). Phenotype D also had increased syndecan-1 levels (*p* < 0.001) and suffered from a higher proportion of EoT (*p* = 0.004).

### 2.4. Endothelial Cell Metabolism in the Four Metabolic Phenotypes

**Figure 3:**
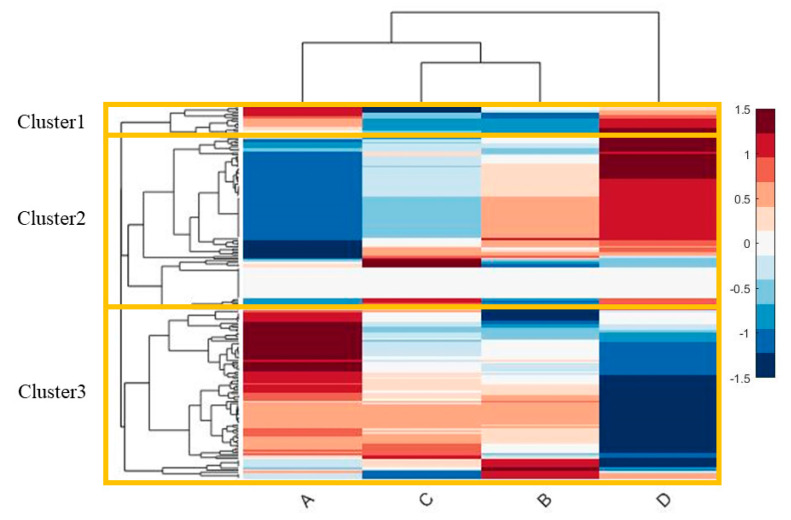
**Calculation of the intracellular cellular activities in phenotypes A, B, C, and D.** Heatmap displays the intracellular activity in phenotypes A–D using GEMs representing each phenotype. **Note:** 190 cellular metabolic tasks were analyzed by GEM EC (iEC3006). All the fluxes are normalized by row, and scaled between −1.5 and 1.5. dendrogram using the Euclidian distance measure and the complete cluster algorithm to combine different cellular activities.

Four GEMs representative of phenotypes (A–D) were reconstructed to investigate the inferred intracellular endothelial metabolism; 190 metabolic function activities were analyzed, and the results were combined into a heatmap (Figure 3). The heatmap displayed three cluster activities; cluster1 included Adenosine triphosphate (ATP) generation from glucose, i.e., combined glycolysis and tricarboxylic acid cycle (TCA) generation from glucose; cluster2 included amino acid degradation, lipids synthesis of arachidonate, palmitoleate, bile acid synthesis, O-glycan metabolism, and mitochondrial Nicotinamide adenine dinucleotide + hydrogen (NADH) generation; cluster3 included synthesis of malonyl-CoA, gluconeogenesis from amino acids, ATP generation from glycolysis, oxidation in the TCA cycle, and redox thioredoxin reductase activity (results from the metabolic function activities are presented in Table S1, see Supplementary Materials).

Both phenotypes A and D were correlated in cluster1 and displayed upregulation. However, Phenotype A and D were anticorrelated in cluster2 and cluster3, were phenotype A was downregulated in cluster2 activity and upregulated in cluster3 activity. Phenotypes B and C had intracellular metabolic activity levels between those of phenotypes A and D (Figure 3).

**Figure 4:**
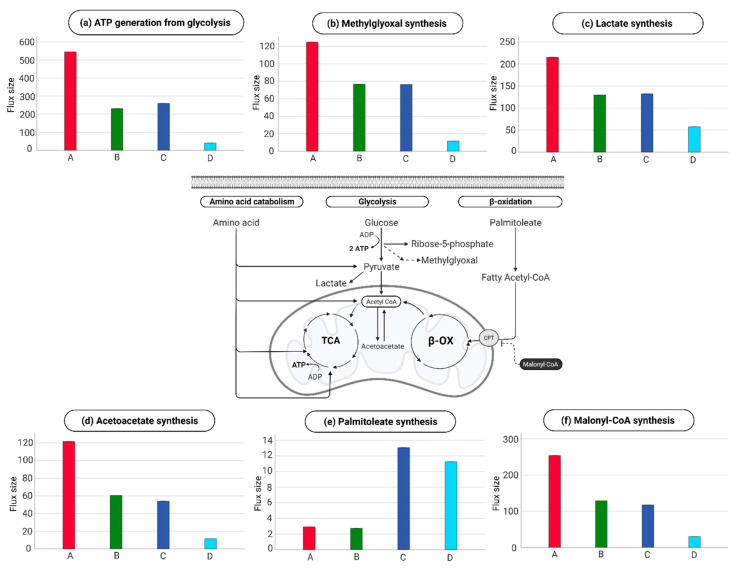
**Cellular activity leading to ATP generation.** (**a**) ATP generation from glycolysis (**b**) Methylglyoxal synthesis (**c**) Lactate synthesis (**d**) Acetoacetate synthesis (**e**) Palmitoleate synthesis (**f**) Malonyl-CoA synthesis. Note: 6 out of the 190 metabolic cellular tasks are displayed in boxplots. The flux size is given in mmol per gDW per h.

Phenotype A had a 13-fold higher rate of ATP generation from glycolysis (Figure 4a), 10-fold higher synthesis of methylglyoxal (Figure 4b), 4-fold higher lactate conversion from glucose (Figure 4c), 1.4-fold synthesis of ribose-5-phosphate (pentose pyruvate pathway), and a 10.5-fold higher rate of Acetoacetate synthesis (Figure 4d) compared to phenotype D. Conversely, phenotype D had increased catabolism of all amino acids (not shown), 3.9-fold increased palmitoleate synthesis (Figure 4e), and an 8.5-fold decrease in the synthesis of malonyl-CoA (Figure 4f).

**Figure 5:**
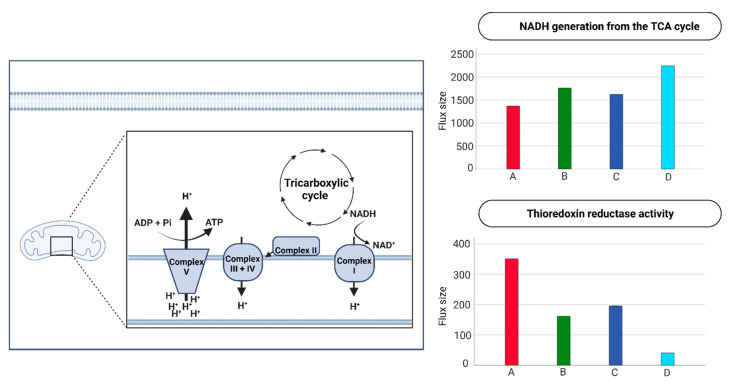
**Activity in the electron transport chain (ECT) within the four phenotypes.** Note: 2 out of the 190 metabolic cellular tasks are displayed in boxplots. The flux size is given in mmol per gDW per h.

When focusing on the TCA-cycle contribution of NADH towards the electrochemical gradient in the mitochondria for ATP-generation, phenotype D had a 1.6-fold increase compared to phenotype A (Figure 5). However, phenotype A had 8.7-fold-increased thioredoxin reductase activity relative to phenotype D (Figure 5.

## 3. Discussion

This study confirmed four extracellular metabolic phenotypes (A, B, C, and D) manifested in patients on arrival at the trauma center, with D associated with injury severity and a comatose state. Thus, phenotype D encompassed 92% of the patients who died within 72 h and had an increased 30-day mortality rate compared to the other three. With regard to the endothelial cell (EC) intracellular metabolic activities phenotypes A and D demonstrated different patterns of cluster 2 and 3, whereas phenotypes B and C had more comparable metabolic profiles.

Phenotype D displayed an extracellular metabolic profile with a distinct branch on the metabolic dendrogram, whereas phenotypes A, B, and C were connected. The principal metabolite distinguishing the four phenotypes was succinic acid, which was elevated in plasma within phenotype D compared to the other phenotypes. Succinic acid has been reported in a cohort of 95 severe trauma patients to be higher in patients who died [14]. Further, the characteristics of phenotype D with enhanced succinic acid levels in plasma, high catecholamine levels, and coupled with glycocalyx shedding, e.g., high levels of syndecan-1, are inline with previous findings [12].

The inferred intracellular metabolic fluxes showed different changes in the EC between phenotypes A and D in cluster 2 and 3 of the investigated metabolic function activities. Both phenotypes had comparable overall glycolytic ATP production (i.e., combined glycolysis and TCA cycle generation of ATP from glucose). However, Phenotype A had increased anaerobic glycolysis, a general characteristic of healthy EC [15,16]. Glycolysis also results in synthesizing glycolic side branches, and in line with phenotype A’s profile, the increased rate in the pentose phosphate pathway leads to nucleotide synthesis and, thereby, sustains the production of macromolecules. Furthermore, glycolysis increases lactate production, which operates as a signaling molecule promoting angiogenesis and cellular adaptation to acidosis [17,18,19,20]. The increased glycolysis and cellular adaptation to an acidic environment and nucleotide synthesis are known as the Warburg effect that was firstly described in cancer cells [21,22]. Despite the beneficial efficacy of producing ATP by glycolysis, an extreme glycolytic turnover result in the production of methylglyoxal; this highly reactive dicarbonyl compound plays a crucial role in advanced glycation end products (AGEs), and its production leads to glycosylation of lipids and proteins. Elevated levels of AGEs are reported after trauma [23] and have been shown to increase EC permeability to macromolecules and further increase cellular reactive oxygen species production leading to cellular stress [24].

Notably, phenotype D had increased amino acid catabolism, which feeds metabolites into the TCA, together with increased palmitoleate synthesis, which β-oxidation can fragment to generate acetyl-CoA for entering the TCA cycle. The β-oxidation in the mitochondria depends on the carnitine palmitoyltransferase transporter for entering fatty acid and is inhibited by malonyl-CoA [25]. In phenotype D, the synthesis of malonyl-CoA was downregulated compared to phenotype A, causing increased β-oxidation. Increased amino acid catabolism and β-oxidation result in high levels of mitochondrial acetyl-CoA and turnover of TCA, thereby generating ATP. Phenotype D’s high levels of circulating catecholamines further damage the endothelial membrane as indicated by syndecan-1 in plasma, which might also contribute to β-oxidation. Phenotype D demonstrated increased ISS compared to the other phenotypes. However, it remains unclear whether the increased amino acid catabolism and β-oxidation towards acetyl-CoA observed in phenotype D is provoked by the trauma or whether it could be genetically determined.

Phenotype D appeared to shift all the generated acetyl-CoA to the TCA cycle, as evidenced by high NADH production. The high NADH generation contributes to high H+ levels and, combined with the reduced thioredoxin reductase activity in phenotype D might lead to pronounced oxidative stress. Increased oxidative stress initiates the peroxidation of fatty acids in the cell membrane, including the mitochondrial membrane [26]. Once initiated, lipid peroxidation initiates a chain reaction of oxidation of the surrounding unsaturated fatty acids leading to membrane disintegration, a central feature of SHINE [27]. Furthermore, mitochondrial membrane damage increases the initiation of new chain reactions by free radicals, provoking intracellular membrane damage in phenotype D. Conversely, phenotype A, with high ATP generation from glycolysis, showed increased formation of acetoacetate from Acetyl-CoA, indicating a higher production of Acetyl-CoA than the TCA turnover demands.

This study had important limitations. First and foremost, we used a limited number of metabolites in plasma to parameterize the EC GEMs and, consequently the results of the intracellular analyses are inferred and need experimental confirmation, i.e., under pathophysiological shock conditions. Further, despite iEC3006 being the most comprehensive GEM of the endothelial cell, expansion of the metabolic coverage to focus on membrane lipids and glycan metabolism is required. Also, we hypothesize that the changes observed in plasma were mainly from the EC since 1 trillion EC are in contact with the circulating blood. We cannot, however, deduct the contribution of metabolites in plasma from other sources, e.g., the liver, muscles, and lipocytes. In addition, we did not have access to pharmacological data prior to admission to the trauma center, which might affect the comatose state of phenotype D. Further, we did not have data on the patients’ genetic variation, e.g., SNP to be integrated into a GEM-based analysis [28] and were, therefore, unable to investigate whether the observed phenotypes reflect a continuum of the shock severity or whether they were genetically determined. Lastly, we only had a 30-day follow-up on mortality.

In conclusion, we confirm that trauma patients appear to have a minimum of four plasma metabolic phenotypes (A–D). Phenotype D was associated with increased mortality and had increased trauma severity scores, but it remains unclear whether increased trauma shock severity drives phenotype D or whether it is genetically determined. The inferred EC intracellular metabolism found two different metabolic patterns between phenotype A and D. Phenotype A produces ATP mainly from anaerobic glycolysis, whereas phenotype D uses catabolism of amino acid combined with β-oxidation of fatty acid towards the TCA, leading to high NADH turnover; this may increase oxidative stress that could be linked to increased mortality.

## 4. Materials and Methods

### 4.1. Setting and Patients

This prospective observational cohort included critically ill adult (≥18 years) trauma patients admitted directly from the scene of injury to the Red Duke Trauma Institute at Memorial Hermann Hospital, Houston, TX between March 2013 and February 2018. The McGovern Medical School approved the study at the UTHealth institutional review board (HSC-GEN-12-0059), and the study was in line with the Declaration of Helsinki.

Blood samples were collected immediately upon patient arrival by on-call research assistants. Informed consent was obtained either from the patient or, if the patient was unconscious, from a legally authorized representative within 72 h after enrollment. A waiver of consent was granted if the patient was discharged or died within 24 h. If consent could not be obtained, the patient was excluded from the study and their blood samples were destroyed.

### 4.2. Patient Selection

Patients were randomly selected for enrollment retrospectively based on the Injury Severity Score (ISS) from a biorepository of more than 6500 patients requiring the highest level of trauma activation. In total, 20 trauma patients with minor and moderate trauma injuries (ISS < 16), 40 trauma patients with serious trauma injury (ISS 16–25), and 39 trauma patients with severe trauma (ISS > 25) were included. The criteria for exclusion were moderate to severe traumatic brain injury, defined as an anatomical injury score for the head > 2, as traumatic brain injury patients have been shown to have a different metabolic profile compared to non-traumatic brain injury trauma patients [29]. The research assistants recorded clinical data in the repository upon admission to the trauma bay or extracted it from medical records and the trauma registry.

### 4.3. Healthy Volunteers

We included 20 healthy volunteers from Denmark to calculate normal plasma metabolic variance to incorporate metabolic patient data into EC-GEM [12]. This was approved by the Regional Ethics Committee (H-4-2009-139) and the Danish Data Protection Agency and was in line with the Declaration of Helsinki.

### 4.4. Analysis of Clinical Characteristics

Statistical analysis was performed using RStudio (version 3.6.3), IBM SPSS statistics (version 25) and MetaboAnalyst (version 5.0). Descriptive patient data are presented as medians with interquartile ranges (IQR) or as a percentage (%). Non-parametric statistical tests (Kruskal-Wallis and Pearson Chi-Square tests) evaluated unpaired group differences as appropriate. A Kaplan-Meier curve displays the survival probability of the different groups.

### 4.5. Sample Preparation

Blood samples were obtained upon hospital admission into 3.2% citrated tubes. Tubes were immediately centrifugated twice at 1800×*g* for 10 min at 5 °C to separate plasma. Plasma was aliquoted and frozen at −80 °C for later analysis.

### 4.6. Enzyme-Linked Immunosorbent Assay (ELISA)

The soluble biomarkers of sympathoadrenal activation (epinephrine and norepinephrine), endothelial glycocalyx (syndecan-1) [30], and endothelial cell soluble thrombomodulin (sTM) [31,32,33] were measured by enzyme-linked immunosorbent assay. Endotheliopathy of Trauma (EoT) was defined by a level of Syndecan-1 ≥ 40 ng/mL [34].

The following manufacturers were used: Epinephrine and norepinephrine (2-CAT ELISA, Labor Diagnostica Nord GmbH Co. & KG, Nordhorn, Germany), Syndecan-1 and sTM (Nordic Biosite, Copenhagen, Denmark).

### 4.7. Mass Spectrometry Analysis

Based on literature and prior work on critical animal models (rats and swine) and critically ill patients, we selected 62 metabolites to be quantified (Table S2) [12,35,36,37,38].

Ultra-high performance liquid chromatography-mass spectrometry was run on a Vanquish system (Thermo Fisher Scientific, San Jose, CA, USA) with a Q Exactive mass spectrometer (HF Hybrid Quadrupole-Orbitrap, Thermo Fisher Scientific, San Jose, CA, USA) [39]. Electrospray ionization (ESI) was performed in negative and positive ionization modes. A QC sample was analyzed in MS/MS mode to identify compounds. The UPLC was performed using a slightly modified version of the protocol described by Catalin et al. [40]; we used chloroform to stop the derivatization reaction. Peak areas were extracted using Compound Discoverer 2.0 (Thermo Fisher Scientific).

Gas Chromatography-Mass Spectrometry (7890B, Agilent) coupled with a quadrupole mass spectrometry detector (5977B, Agilent) and controlled by ChemStation (Agilent) was used to detect amino and non-amino organic acids. Raw data were converted to netCDF format using Chemstation (Agilent) before being imported and processed in Matlab R2018b (Mathworks, Inc., Natrick, MA, US) using the PARADISe software [41]. The mass spectrometry analysis was run by MS-Omics (https://www.msomics.com/, accessed on 7 November 2022).

### 4.8. Analysis of Mass Spectrometry Data

Eight metabolites had an undetectable value in more than 30% of the samples and were consequently excluded from further analysis (Table S2). For the remaining 54 metabolites, less than 2% of the values were missing. Missing values were imputed using the Missforest package [42] in R, which applies a random forest approach to impute values, minimally altering the statistical characteristics of the metabolite.

The quantified metabolic data were normalized by log2 transformation and Pareto scaling to create Principal Component Analysis (PCA), Partial Least-Squares Discriminant Analysis (PLS-DA), and a heatmap with a hierarchical clustering dendrogram using the Euclidian distance measure and the complete cluster algorithm. Further, fumaric acid was removed from the metabolic statistical analysis because it correlated 1:1 with malic acid.

PCA plots were used to detect potential outliers. Four patients were considered outliers due to a metabolic profile outside of the 95% PCA 1-2 confidence interval (one patient in the 3-dimensional plot PCA 1-3) (Table S3). Also, the four patients’ metabolic profiles could not be explained by an extreme clinical presentation and were therefore removed from further analysis, leaving 95 subjects for the subsequent analysis

### 4.9. Analysis of Data with iEC3006 Genome-Scale Metabolic Model

The GEM EC (iEC3006) is the most extensive genome-scale metabolic model of the endothelial cell and includes 2035 genes and 3006 reactions involving a total of 2114 metabolites [43]. Genome-scale metabolic models (GEMs) provide a convenient platform for the integration and analysis of case-specific plasma metabolomics data enabling to infer the metabolic flux profile associated to a given phenotype.

This study generates GEMs specific to each of the four phenotypes by parameterizing iEC3006 with each phenotype’s mean patient plasma metabolic profiles by using the COBRA Toolbox in Matlab R2017b (26). First, constraints on uptake and secretion of metabolites were determined by the upper and lower quartiles of their respective transport reaction flux distributions as determined by random sampling flux analysis of the baseline version of iEC3006.

To determine the normal metabolic variances, we included 20 healthy volunteers. Mean fold changes (patient phenotype/healthy volunteers) from the plasma metabolomics data set were applied to the upper and lower quantiles of each transport reaction to define the uptake and secretion in the patient phenotype models; if necessary, reactions were relaxed to obtain a feasible model (Tables S4 and S5). Consequently, the phenotype models were constructed (https://github.com/HHEN0042/Endothelial-cell-phenotypes.git, accessed on 7 November 2022).

### 4.10. Validation of GEMs Reconstruction

Cross-validation analysis was performed to evaluate the accuracy of the developed GEMs of phenotypes A, B, C, and D [44]. Each phenotype GEM model was constructed where one of each constrained exchange reaction was left out each time, and the corresponding population of possible flux values for each unconstrained exchange reaction was calculated by applying a sampling analysis. Next, to determine the goodness of phenotypes model predictions, the population of solutions from the unconstrained models were compared to each of the initial phenotype GEMs models, i.e., phenotypes A, B, C, and D, and the significance was calculated by *t*-test and FDR adjusted.

The validation of GEMs phenotype A, B, C, and D predicted 90.6% of the metabolite uptake/secretion rates with an associated *p*-value < 0.05 with high accuracy; R^2^ equal to 0.999, 0.997, 0.999, and 0.969, respectively (Table S6).

### 4.11. Inferring Metabolic Task Activity in Trauma Groups via GEMs

We combined GEM-based flux balance analysis manually curated cellular metabolic tasks to investigate differences within the intracellular metabolism between the phenotypes [45,46]. In total, 190 metabolic cellular tasks, each describing the synthesis/degradation of different metabolites from/to different metabolic sources/products, were investigated (Supplementary Table S7) [46]. As result an optimal flux value indicating the activity of each metabolic task was calculated for each trauma group. Next, the metabolic task activities were integrated into a heatmap, normalized by row, and scaled to values between −1.5 and 1.5, with a dendrogram using the Euclidian distance measure and the complete cluster algorithm to combine different cellular activities.

## Figures and Tables

**Table 1:** Demographics, admission vitals, transfusion, and outcome for 95 critically ill trauma patients.

		Phenotype D	Phenotype C	Phenotype B	Phenotype A	
		(n = 21)	(n = 24)	(n = 17)	(n = 33)	*p*-Value
**Demography**						
Age	Years	43.0 [28.0, 50.0]	36.0 [30.0, 46.0]	45.0 [37.0, 54.0]	50.0 [41.0, 60.0]	**0.021**
Sex	Male (%)	19 (90.5%)	15 (62.5%)	13 (76.5%)	23 (69.7%)	0.176
Race	Race [n (%)]	White = 4 (19.0%) African American = 7 (33.3%) Hispanic = 7 (33.3%) Asian = 0 (0%) Other = 3 (14.3%)	White = 8 (33.3%) African American = 7 (29.2%) Hispanic = 6 (25.0%) Asian = 0 (0%) Other = 3 (12.5%)	White = 5 (29.4%) African American = 1 (5.9%) Hispanic = 7 (41.2%) Asian = 2 (11.8%) Other = 2 (11.8%)	White = 20 (60.6%) African American = 10 (30.3%) Hispanic = 2 (6.1%) Asian = 0 (0%) Other = 1 (3.0%)	**0.004**
BMI	Score	26.7 [25.0, 30.1]	27.1 [24.8, 28.8]	29.5 [28.1, 33.7]	26.8 [24.7, 31.3]	0.229
**Injury type and severity**						
ISS	Score	34.0 [25.0, 45.0]	25.0 [9.75, 29.0]	25.0 [22.0, 29.0]	21.0 [9.00, 25.0]	**<0.001**
AIS Head	Score	0 [0, 0]	0 [0, 0]	0 [0, 0]	0 [0, 0]	0.175
AIS Face	Score	0 [0, 0]	0 [0, 0]	0 [0, 1]	0 [0, 0]	**0.027**
AIS Thorax	Score	3.00 [3.00, 4.00]	1.50 [0, 3.00]	2.00 [0, 3.00]	0 [0, 3.00]	**0.010**
AIS Abdomen	Score	4.00 [3.00, 4.00]	0 [0, 2.25]	0 [0, 2.00]	0 [0, 2.00]	**0.001**
AIS Extremity	Score	3.00 [0, 4.00]	2.50 [0, 3.00]	0 [0, 2.00]	0 [0, 0]	**0.011**
AIS External	Score	1.00 [1.00, 1.00]	1.00 [0.750, 2.75]	1.00 [0, 5.00]	1.00 [0, 2.00]	0.835
GCS	Score	3.00 [3.00, 13.0]	14.5 [3.00, 15.0]	15.0 [3.00, 15.0]	15.0 [7.00, 15.0]	**0.028**
**Admission median blood pressure**						
SBP	mmHg	98.0 [84.0, 114]	111 [102, 140]	119 [102, 132]	132 [118, 142	**<0.001**
Heart rate	Bpm	112 [98.0, 120]	101 [92.0, 111]	93.0 [84.0, 109]	96.0 [73.5, 115]	0.171
**Blood variables**						
Base excess	mEq/L	−13.0 [−16.0, −9.00]	−6.00 [−8.25, −2.00]	−3.00 [−8.00, −2.00]	−5.00 [−7.00, −2.00]	**<0.001**
Lactate	mg/dL	9.80 [6.85, 12.9]	3.70 [2.75, 4.45]	3.70 [3.10, 5.70]	2.30 [1.60, 3.55]	**<0.001**
Glucose	mg/dL	229 [199, 324]	145 [119, 165]	173 [145, 213]	134 [115, 196]	**<0.001**
**Transfusions pre-hospital**						
Transfused pre-hospital?	Yes [n (%)]	6 (28.6%)	2 (8.3%)	3 (17.6%)	8 (24.2%)	0.329
if yes:						
RBC	Units	0.500 [0, 1.00]	0.500 [0.250, 0.750]	0 [0, 0.500]	1.00 [1.00, 1.50]	0.056
Plasma	Units	1.00 [1.00, 1.00]	0.500 [0.250, 0.750]	1.00 [0.500, 1.00]	1.00 [0.750, 1.25]	0.704
Whole blood	Units	0 [0, 0]	0.500 [0.250, 0.750]	0 [0, 0.500]	0 [0, 0]	0.336
**Transfusions after admission**						
Transfused within 4 h?	Yes [n (%)]	19 (90.5%)	13 (54.2%)	8 (47.1%)	15 (45.5%)	**0.007**
if yes:						
RBC	Units	12.0 [5.00, 32.0]	2.00 [1.00, 5.00]	1.50 [0.750, 2.25]	2.00 [2.00, 3.50]	**<0.001**
Plasma	Units	14.0 [4.00, 32.0]	4.00 [1.00, 8.00]	1.00 [1.00, 2.25]	3.00 [1.00, 4.00]	**<0.001**
Platelets	Units	12.0 [0, 21.0]	0 [0, 6.00]	0 [0, 0]	0 [0, 0]	**0.007**
Transfused within 24 h?	Yes [n (%)]	19 (90.5%)	16 (66.7%)	11 (64.7%)	21 (63.6%)	0.156
if yes:						
RBC	Units	14.0 [5.00, 35.0]	2.00 [0, 5.75]	1.00 [0, 2.00]	2.00 [0, 3.00]	**<0.001**
Plasma	Units	17.0 [4.50, 35.0]	5.00 [1.75, 10.3]	2.00 [1.00, 4.00]	4.00 [1.00, 9.00]	**0.001**
Platelets	Units	12.0 [0, 24.0]	0 [0, 1.50]	0 [0, 0]	0 [0, 0]	**0.003**
**Outcome**						
Mortality (<24 h)	n (%)	11 (52.4%)	0 (0%)	0 (0%)	1 (3.0%)	**<0.001**
Mortality (<72 h)	n (%)	12 (57.1%)	0 (0%)	0 (0%)	1 (3.0%)	**<0.001**
Mortality (<30 days)	n (%)	16 (76.2%)	4 (16.7%)	5 (29.4%)	10 (30.3%)	**<0.001**

Median (IQR) or n (%). Calculation of the *p*-value on phenotype D compared to the other phenotypes. *p*-values ≤ 0.050 are shown in bold. BMI, body mass index; AIS, abbreviated injury score; GCS, Glasgow coma score; SBP, systolic blood pressure; RBC; red blood cells.

**Table 2:** Enzyme-linked immunosorbent assay measurement for 95 critically ill trauma patients.

		Phenotype D	Phenotype C	Phenotype B	Phenotype A	
ELISA		(n = 21)	(n = 24)	(n = 17)	(n = 33)	*p*-Value
Epinephrine	pg/mL	2240 [1110, 4320]	230 [99.4, 710]	262 [211, 363]	271 [63.1, 426]	**<0.001**
Norepinephrine	pg/mL	3460 [1920, 13,100]	741 [427, 1400]	1180 [492, 3000]	1180 [604, 1680]	**<0.001**
sTM	ng/mL	7.06 [6.01, 11.2]	5.93 [5.01, 7.09]	6.32 [5.15, 10.5]	6.46 [5.15, 9.28]	0.291
Syndecan-1	ng/mL	190 [120, 198]	42.3 [24.5, 120]	49.5 [37.2, 166]	34.4 [22.5, 101]	**<0.001**
EoT	Yes [n (%)]	19 (90.5%)	13 (54.2%)	10 (58.8%)	16 (48.5%)	**0.016**

Medians (IQR) or n (%). Calculation of the *p*-value is on phenotype D compared to the other phenotypes. *p*-values ≤ 0.050 are shown in bold. sTM; soluble thrombomodulin, EoT; endotheliopathy of trauma.

## Data Availability

https://github.com/HHEN0042/Endothelial-cell-phenotypes.git, accessed on 7 November 2022.

## Supplementary Materials

The following supporting information can be downloaded at: https://www.mdpi.com/article/10.3390/ijms24032257/s1.

## Abbreviations

AGEs	Advanced glycation end products
ATP	Adenosine triphosphate
EC	Endothelial cell
EC-GEM	Genome-scale metabolic model of the endothelial cell
EoT	EoT Endotheliopathy of Trauma
GEMs	Genome-scale metabolic models
ISS	Injury Severity Score
NADH	Nicotinamide adenine dinucleotide + hydrogen
PCA	Principal Component Analysis
PLS-DA	Partial Least-Squares Discriminant Analysis
SHINE	Shock-induced endotheliopathy
sTM	Soluble thrombomodulin
TCA	Tricarboxylic acid cycle
